# Principles and Practice of Obstetric Medicine

**Published:** 1836-07-01

**Authors:** 


### PRINCIPLES AND PRACTICE OF OBSTETRIC MEDICINE, &c. &c. By Dr. *Davis*. Parts XXIV. and XXV.

FEW elementary works have come under our notice containing so much original matter as this of Dr. Davis;—and it is on this account that we are tempted, from time to time, to extract valuable matters from its pages. In a former number we gave an account of that dreadful and disgusting complaint—NYMPHOMANIA—we now proceed to other uterine affections—less frightful, but much more common than the one in question.

*Malposition.* The principal malpositions of the unimpregnated uterus are prolapsion, anteversion, and extra-abdominal protrusion. Prolapse is by far the most frequent. To express the three stages or degrees of uterine subsidence, Dr. D. applies the term DILAPSION to the first stage, or simple bearing-down of the womb on an inferior portion of the vagina:—PROLAPSION to the second stage, when part of the uterus is engaged within the vaginal orifice—and PROCIDENTIA to the third, when the entire uterus is protruded out of the pelvic cavity, carrying with it a part or the whole of the vagina inverted.

1. *First Stage, or Dilapsion.* In this, the uterus descends two or three inches below its natural level, so as to press upon and annoy the vaginal parietes. The effects are, a sense of inconvenience and irritation in the vagina—an actual shortening of the vaginal tube—a leucorrhœal discharge —impeded or painful micturition—sometimes tenesmus.

2. *Prolapsion.* Here the vaginal portion of the uterus may be seen to project through the os externum, and make its appearance within or even beyond the level of the labia majora.

"The womb in this case occupies the inferior part of the cavity and an anterior portion of the outlet of the pelvis; and its body is almost every where invested by a double covering of vaginal structure; viz. first by the superior portion of it, with the extremity of which it is immediately adherent, and which it therefore cannot fail to take along with it, reflected and inverted towards the outlet of the pelvis; and secondly, by so much of its middle and inferior portions as may remain unreflected, and continue to retain their accustomed relations to the parietes of the pelvis at and near its outlet, and to the tissues naturally connected with the lower part of the pelvic cavity." 522.

To the symptoms before-mentioned, are now added dragging pains at the groins—aching pain in the back, in the sacral, and vesical regions—sometimes in the thighs. The recumbent posture gives relief to these symptoms.

3. The PROCIDENTIA, or complete prolapse, need not be described, as it is visible to the naked eye.

The *proximate* cause of these different degrees, the author considers to be a reduced power of the suspensory ligaments, generally accompanied by a relaxed state of the vaginal parietes.* The predisposing causes depend on ori-

---

* It will be seen in next article, that Dr. Hamilton, of Edinburgh, differs from Dr. D. on this point.—EDS.

ginal slenderness and delicacy of the ligamentous and membranous tissues destined for suspension of the uterus—feeble health—many puerperal trials.

*Occasional Causes.* In nine cases out of ten, prolapsus uteri is an early consequence of the puerperal state, generally from getting up too soon, and resuming her wonted avocations after parturition. Several excellent illustrations are given by the author from his own practice or that of others. Next to these causes are prolapsions and protrusions of the uterus, *during gestation*, imputable to the weight of the womb in that state, and before quickening.

On the diagnosis of the different stages, the author descants very fully; but we must refer to the work itself on this point, with the exception of the following case.

" A considerable protrusion of the rectum was once mistaken for a case of procidentia with inversion of the uterus. The tumour first presented itself contemporaneously with the birth of a large child. The portion of protruded intestine was of such a magnitude and so tensely distended, as to have had the effect of concealing all the passages from the pelvis. The author, however, soon learnt that the after-birth had not been removed, and he found the placental portion of the umbilical cord prolapsing from between the person of the patient anteriorly and the mass of inverted intestine-looking substance which occupied the space, as already described, between the nates and the superior parts of the thighs. The umbilical cord was firmly taken hold of by the left hand, whilst the right was gradually and very cautiously carried up along it into the vagina, which was found widely developed and empty. At that moment the placenta could not be reached without causing more pressure to be made upon the tumour than might be convenient, or was at all necessary. There was no discharge of blood, and the uterus could be distinctly recognised through the parietes of the hypogastrium. These facts went incontestably to prove that the placenta was still attached to the uterus, and that the uterus itself occupied its proper situation, under its then circumstances, within the abdominal and pelvic cavities. In its feel and form, and character of tissue, the protruded body furnished unequivocal proofs of its proper nature and source; to the latter of which it was indeed in a few minutes very satisfactorily traced; and in about half an hour afterwards it was equally satisfactorily reduced." 548.

*Treatment of Descents of the Uterus.* Here, more perhaps than in any other complaint, prevention is better than cure. The causes, in nine cases out of ten, are, the too early resumption of ordinary occupations after puerperal confinements.

" The uterus often contracts in the course of a few hours after delivery to a volume sufficiently small to admit of its easy delapsion into the pelvic cavity, provided the patient were allowed or encouraged to assume positions which might favour such a descent; whereas, at that early period, little or no resistance could be expected to be opposed to such a tendency by reason of the state of extreme relaxation and development both of the uterine ligaments and of the parietes of the vagina. Experience therefore has long established the propriety, and indeed the necessity, of causing women, during the period in question, LITERALLY TO LIE IN; that is, to use, more or less exclusively, at that time, the recumbent position, and also to take the benefit of that position within doors, or perhaps more expressly within their own chambers." 548.

There is no precise measure of time for the allotment of this duty. The uterus is not supposed to recover its proper size and weight, in less than six weeks after delivery. But the suspensory ligaments and other structures by which the uterus is connected with the pelvis, generally recover their tone in half that time or less. Strict confinement to bed may usually be limited to the first eight or ten days after confinement—the remainder of the three weeks being allotted to very gentle movements in the patient's chambers.

The indications of cure are three—reduction of the descent, if necessary, by manual assistance—retention of the organ in its proper place—attention to the general health. In many incipient cases, descents of the uterus are remedied by attention to the general health alone, or with some topical applications. Active tonics, preceded or accompanied by alteratives, are the best means to be employed. Where the vagina is in a relaxed state, astringent injections are useful.

When, however, the prolapsion is chronic, the pessary is almost inevitable. Dr. D. thinks that the sponge-pessary is the best, and gives a long description of an ingenious contrivance for that purpose, which would not be understood without the plates. Where the sponge cannot be managed, the next best substitute is the ring pessary. The best are those which are made of light wood.

Our author, after a long enumeration of the various kinds of pessaries that have been invented, proceeds to a consideration of the third and last degree of descent—viz. that of its reduction. When this form of malposition is only partial, the prolapsed organ frequently retires into the pelvis spontaneously, in the horizontal posture—or, if not, the patient herself is able to effect the reduction with her own fingers. A cloud of cases are next cited by Dr. Davis, from old and recent writings which must be consulted in the work itself.

